# The Influence of Oxidized Imino-Allantoin in the Presence of ^OXO^G on Double Helix Charge Transfer: A Theoretical Approach

**DOI:** 10.3390/ijms25115962

**Published:** 2024-05-29

**Authors:** Boleslaw T. Karwowski

**Affiliations:** DNA Damage Laboratory of Food Science Department, Faculty of Pharmacy, Medical University of Lodz, ul. Muszynskiego 1, 90-151 Lodz, Poland; boleslaw.karwowski@umed.lodz.pl

**Keywords:** oxidized imino-allantoin, 7,8-dihydro-8-oxo-2′-deoxyguanosine, clustered DNA damage, glycosylase, BER, charge transfer, DFT

## Abstract

The genome is continuously exposed to a variety of harmful factors that result in a significant amount of DNA damage. This article examines the influence of a multi-damage site containing oxidized imino-allantoin (^OX^Ia) and 7,8-dihydro-8-oxo-2′-deoxyguanosine (^OXO^dG) on the spatial geometry, electronic properties, and ds-DNA charge transfer. The ground stage of a d[A_1_^OX^Ia_2_A_3_^OXO^G_4_A_5_]*d[T_5_C_4_T_3_C_2_T_1_] structure was obtained at the M06-2X/6-D95**//M06-2X/sto-3G level of theory in the condensed phase, with the energies obtained at the M06-2X/6-31++G** level. The non-equilibrated and equilibrated solvent-solute interactions were also considered. Theoretical studies reveal that the radical cation prefers to settle on the ^OXO^G moiety, irrespective of the presence of ^OX^Ia in a ds-oligo. The lowest vertical and adiabatic ionization potential values were found for the ^OXO^G:::C base pair (5.94 and 5.52 [eV], respectively). Conversely, the highest vertical and adiabatic electron affinity was assigned for ^OX^IaC as follows: 3.15 and 3.49 [eV]. The charge transfers were analyzed according to Marcus’ theory. The highest value of charge transfer rate constant for hole and excess electron migration was found for the process towards the ^OXO^GC moiety. Surprisingly, the values obtained for the driving force and activation energy of electro-transfer towards ^OX^Ia_2_C_4_ located this process in the Marcus inverted region, which is thermodynamically unfavorable. Therefore, the presence of ^OX^Ia can slow down the recognition and removal processes of other DNA lesions. However, with regard to anticancer therapy (radio/chemo), the presence of ^OX^Ia in the structure of clustered DNA damage can result in improved cancer treatment outcomes.

## 1. Introduction

Genetic information is encoded in the DNA matrix, which consists of3.2 × 10^9^ nucleotides. The distribution of nucleobases in DNA has been found as follows: guanine (G) 20%, cytidine (C) 20%, adenine (A) 30%, and thymine (T) 30% [1]. According to the isochore theory, the double helix is a mosaic of different levels of GC ranging from 37% to 52% [2]. This fragile material, in which the secret of life is written, is continuously exposed to harmful factors, both exocellular and endocellular [3]. Regardless of their nature—whether physical or chemical—their interaction with the genome can induce DNA damage, which, if left unrepaired, can lead to mutations and subsequently the onset of carcinogenesis. Over the course of 24 h, in each of the 10^14^ human cells, 10^5^ DNA lesion events take place [4]. Of all nucleobases, guanine exhibits a lower oxidation potential of 1.29 [V] vs. NHE in comparison to adenine (1.42 [V]), thymine (1.7 V), and cytidine (1.6 V) [5]. As a result, dG is the most susceptible to one-electron oxidation. Reactive oxygen/nitrogen species such as the hydroxyl radical (^●^OH) and singlet oxygen (^1^O_2_) react preferentially with dG depending on its location and the neighboring nucleotides in ds-DNA [6]. In the vast majority of nucleobase lesions, 7,8-dihydro-8-oxo-2′-deoxyguanosine (^OXO^dG) has been found to be the most frequent.

**Figure 1:**
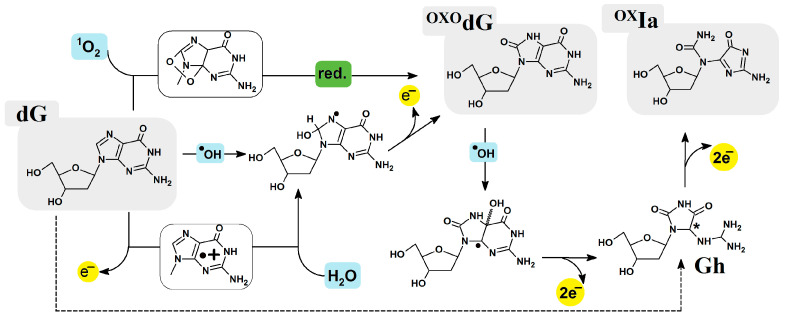
Graphical representation of oxidized imino-allantoin formation via 2′-deoxyGuanosine oxidation paths [9,10]. * chiral center 4*R* or 4*S*.

It is commonly accepted that ^OXO^dG, in a cell, is present at a level of 8 × 10^−6^ bases. Moreover, its ionization potential has been assigned as 0.75 [eV], which makes it much more predisposed than dG towards further oxidation [7]. As presented in Figure 1, dG can transform into ^OXO^G, guanidinohydantoin (Gh), and finally into oxidized imino-alantoin (^OX^Ia) in the two-, four-, and six-electron oxidation cascade, which is triggered by one-electron abstraction or the addition of a hydroxyl radical. The hydantoin lesions are less abundant by one order of magnitude than ^OXO^G [8,9]. During evolution, to avoid unwanted changes to genetic information, cells developed a DNA damage repair response system [10]. The above-presented DNA lesions are removed from the genome by base excision repair (BER), which is initiated by a mono- or bifunctional glycosylase [11]. These enzymes can recognize, in the vast majority of nucleosides, the lesioned moiety and activate other BER proteins for subsequent action. However, failure in the guanosine DNA lesion recognition/repair system causes transversion in the genome GC→AT or GC→CG [12]. Recently, it has been proposed that glycosylases such as MutYh utilize electron transfer through the double helix to detect DNA damage very effectively [13]. This process has been discussed in the context of isolated damage; however, clustered lesions consisting of the final dG oxidation product, i.e., Ia^OX^, have yet to be taken into consideration. Both diastereomers of guanidinohydantoin can easily rearrange to iminoalantoin (at pH > 8.2) and finally to ^OX^Ia [14]. Unlike spiroiminodihydantoin (Sp), which primarily forms in single-stranded DNA, Gh predominantly forms in ds-DNA [15,16,17]. The amount of guanidinohydantoin was determined at the limit of detection, i.e., 1–7 per 10^8^ nucleosides, using HPLC-ESI-MS/MS [18]. Further Gh oxidation leads to its oxidation, with subsequent conversion to ^OX^Ia [8]. It should be noted that six-electron oxidation is a rare event under typical physiological conditions. However, during anticancer therapy treatment (e.g., ionization radiation), the occurrence of six-electron oxidation can significantly increase, which can promote ^OX^Ia formation. Moreover, identification and quantification of the second oxidation products, such as Gh, Sp, and ^OX^Ia, pose serious challenges for analytical techniques. This is because of their instability, high hydrophilicity, and the fact that they appear as a mixture of diastereomers [19]. Even in low abundance, Gh and its oxidized derivative can play a significant role in mutagenesis. It has been found that unlike ^OXO^dG, which yields 3% of G→T transversion, Gh results in almost 100%, with G→C preferences [20,21,22]. Barrows et al. have found that imino-alantoine forms predominantly the base pair with dGTP, which leads to G→C transversion [14]. Therefore, even though ^OX^Ia appears in the genome in low amounts, its presence can have a significant impact on genetic information stability, particularly in the context of defects in the NEIL family of glycosylases [23,24]. Furthermore, the presence of ^OX^Ia in the structure of the double helix can lead to a protein communication process via charge transfer [25]. It has been found that after electron loss, the binding of MutYh to ds-DNA increases 1000 times [13,26,27]. If no reduction takes place, it can hinder other proteins’ movement, such as OGG1. Until now, the 4[FeS] cluster has not been found in the structure of OGG1, which would appear to indicate that this protein scans ds-DNA in classical mode, i.e., base by base. However, the presence of iron and sulfur is not necessary for the glycolytic activity of MutY. This does not rule out the possible presence of a 4[FeS] cluster in the OGG1 structure, though. The above indicates that the cumulation of DNA damage over a short distance renders the damage repair process difficult with a longer repair time and decreased fidelity [28]. On the other hand, it should be mentioned here that during radio/chemo or combined therapy, the number of clustered DNA lesions (CDL) increases, making the DNA repair process much more complicated and laborious. It should be pointed out here that CDLs are generated not only within (targeted) cancer cells but also in healthy ones. In light of this, the recognition and repair processes of CDLs are an important consideration in terms of the effectiveness and safety of anticancer therapy [29,30]. Hence, in this article, the influence of oxidized imino-alantoin on the charge transfer through ds-DNA in the presence of ^OXO^dG has been investigated and is discussed theoretically at the M06-2x/6-31++G** level of theory in the aqueous phase using equilibrated and non-equilibrated solvent-solute interaction.

## 2. Results

**Figure 2:**
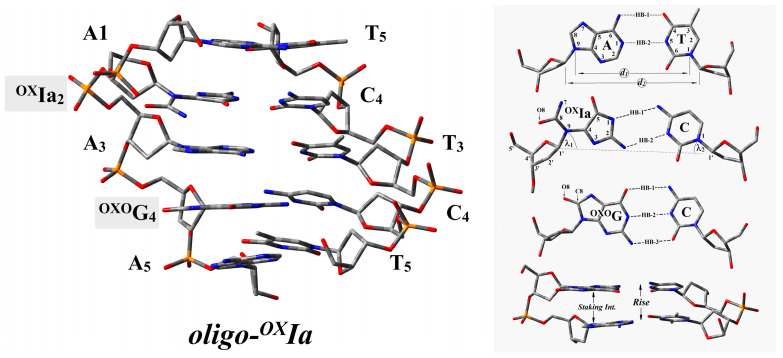
The structure of double-stranded oligonucleotide (***oligo-^OX^Ia***) containing cluster DNA damage site optimized on M06-2x/D95**:M06-2x/sto-3G level of theory in aqueous phase and graphical representation of geometrical parameters hydrogen bonds (HB), distances (*d*_1_ and *d*_2_), angels (*λ*), distance between base pairs within a dimmer (*Rise*), staking interaction, and suitable nucleoside atom numbering.

A clustered lesion is defined as two or more DNA lesions per one or two helix turns. This type of damage distribution in the genome poses a serious challenge for cell repair systems. The first step, not only in BER but also in all cases of DDR (DNA damage response), is target (lesion) recognition, which restricts and determines the rate of the whole process. With this in mind, the short double-stranded (ds) oligonucleotide containing ^OX^Ia and ^OXO^dG in the purine strand, i.e., d[A_1_**^OX^Ia_2_**A_3_**^OXO^G_4_**A_5_]*d[T_5_C_4_T_3_C_2_T_1_] (denoted as *oligo-^OX^Ia*), was taken into consideration as a CDL model. The structures of *oligo-^OX^Ia* in their neutral, anionic, and cationic forms were optimized at the M06-2x/D95**:M06-2x/sto-3G level of theory (Figure 2). The complexity of the analyzed system necessitated the application of the ONIOM (Our Own N-layered Integrated Molecular Orbital and Molecular Mechanics) strategy, thus reducing computational time costs. Since in living cells, all biochemical reactions, including charge transfer, take place in the aqueous phase, the polarizable continuum model (PCM) was applied. The double helix spatial geometry is stabilized by two factors: hydrogen bonds (HB) and staking interaction. The energy of both non-covalent interactions depends on the mutual position between complementary bases (HB) and the distance between base pairs within the formed dimer (*Rise*). Figure 2 presents a schematic representation of the discussed parameters. Structural analysis of the obtained geometry reveals that ^OX^Ia has negligible influence on the geometry of the double helix. Even though the purine ring was rearranged, converting to oxidized imino-allantoin in the base pair formed with cytidine (^OX^Ia_2_::C_4_), the presence of two hydrogen bonds was still observed. Furthermore, the length and energy of the remaining HBs were noted at the same level as those obtained for the corresponding native ds-oligo (Table 1) [31,32]. Additionally, the distances *d*_1_ and *d*_2_, which describe the spatial arrangement of complementary bases, were observed at the levels of 9 Å and 10.8 Å, respectively. Only the value of angle λ_1_ was significantly lower than that obtained for other BPs, i.e., 39.2° versus 53° as the results of the imidazole ring opening in the ^OX^Ia moiety. The lack of significant spatial geometry distortion forced by ^OX^Ia is confirmed by the local base pair step parameter *Rise* [Å] and stacking interaction energy *E_ST_* [kcal]. The distances between the two base pairs in the dimer (*Rise*) formed by ^OX^Ia_2_::C_4_ and A_1_::T_5_ or A_3_::T_3_ were as follows: 3.0 [Å] and 2.99 [Å], respectively. The above corresponds to 14.77 and 15.90 kcal of stacking interaction energy in the discussed system. It should be pointed out for reference that the native *ds-oligo* parameters were as follows: *Rise*: 3.21/2.96 [Å]and *E_ST_*: 14.56/13.59 [kcal] for G_2_::C_4_ and A_1_::T_5_/A_3_::T_3_, respectively [33].

### 2.1. The Influence of ^OX^Ia as Part of a CDL on Double Helix Electronic Properties

Because of the (parallel) stacked nucleobase’s similarity to graphene, DNA has been considered a nanowire for the last two decades [34]. On the other hand, enzymes like glycosylases have utilized charge transfer to scan the double helix toward damage identification/location almost from the beginning of evolution [25]. The above indicates that a DNA lesion, especially a CDL, can modify the charge transfer. It has been recently postulated that electrons are able to migrate through ds-DNA at a distance of thousands of base pairs [13]. Therefore, it can be predicted that the appearance of additional electrons or the loss of electrons in the system should influence the double helix structure. Because of this, the presence of a CDL in *oligo-^OX^Ia* can change its “global” electronic properties [35]. In these studies, computation power constraints only allowed consideration of the ds-pentamer in its adiabatic, cationic, neutral, and anionic forms in the aqueous phase as a native DNA environment.

**Table 1:** The structural local base pair parameters: *Rise* and *d_1(C1′…C1′)_* and *d_2(C9…C1)_* in [Å], *λ*_1_ and *λ*_2_ in [°] according to the standard DNA reference frame of ***oligo-^OX^Ia***, presented in Figure 2. The HB lengths are given in [Å] (HB-1: Ade(N1), Thy(N3) and Gua(O6), Cyt(N4); HB-2: Ade(N6), Thy(O4) and Gua(N1), Cyt(N3); HB-3: Gua(N2), Cyt(O2), for ^OX^Ia_2_C_4_: HB-1 (Cyt(N4), ^OX^Ia(N1), HB-2 Cyt(N3), ^OX^Ia(N2)). The HBs and stacking energies (*E_HB_* and *E_ST_*) are given in [kcal].

Base Pair	HB-1	HB-2	HB-3	*E_HB_*	*λ*_1_	*λ*_2_	*d*_1_	*d*_2_	Base Pair Dimer	*Rise*	*E_ST_*
**A_1_T_5_**	2.80	2.99		10.84	51.5	52.0	10.7	8.83	**A_1_T_5_|^OX^Ia_2_C_4_**	3.00	14.77
**^OX^Ia_2_C_4_**	3.00	2.99		12.40	42.5	55.5	11.4	9.37	**^OX^Ia_2_C_4_|A_3_T_3_**	2.99	15.90
**A_3_T_3_**	2.84	2.89		10.74	48.7	50.7	10.8	8.95	**A_3_T_3_|^OXO^G_2_C_4_**	2.97	16.70
**^OXO^G_2_C_4_**	2.86	2.88	2.86	17.54	52.1	55.5	10.8	9.00	**^OXO^G_2_C_4_|A_5_T_1_**	2.95	15.19
**A_5_T_1_**	2.82	2.99		10.51	53.7	56.1	10.5	8.85			
	Parameters calculated for ideal mode [35]										
**AT** [16]	2.96	3.05		10.81	54.5	54.5	10.7	8.58	**GC||AT** [17]	2.96	14.56
**GC** [16]	3.00	3.00	2.87	17.23	54.2	54.5	10.8	9.01	**AT||GC** [17]	3.31	13.59

To clarify the above-mentioned hypotheses, a comparative analysis was conducted to compare neutral versus anion and neutral versus cation. The analysis revealed that there was a higher structural fluctuation following the acceptance of an extra electron in the system than following the loss of an electron. As the parameter for the above analysis, the RMSD was chosen in Å^2^. In both cases, a higher RMSD value was observed in the case of the sugar-phosphate backbone than for base pair (BP) leaders, as shown in Table 2. The above results suggest significant changes in electronic properties between positively and negatively charged molecules. Because all macromolecules existing in the cell were immersed in an aqueous environment, the non-equilibrated and equilibrated models of solvent-solute interaction were applied. The above, following the work of Sevilla et al., suitably described the influence of solvents on the electronic properties of macromolecules [36]. As expected, during the solvent equilibration process and structure relaxation, the calculated ionization potentials (IP) of *oligo-^OX^Ia* decreased in the following order: VIP^NE^ > VIP^EQ^ > AI (Table 2). Moreover, the IP was found to be lower than that assigned for native ds-oligo in all cases, which indicates that the presence of ^OXO^dG, as part of clustered lesions, plays a significant role. Contrary to the above, the adoption of an electron by *oligo-^OXO^Ia* leads to anion formation, firstly vertical, then, after geometry relaxation, adiabatic. The results of the performed computation reveal that the appearance of ^OX^Ia in ds-oligo significantly increases electron affinity (EA) in comparison to *oligo-N* in all discussed cases, i.e., VEA^NE^ < VEA^EQ^ < AEA.

**Figure 3:**
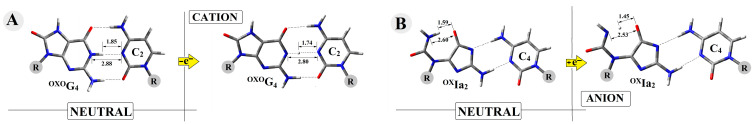
Graphical representation of proton charge transfer after neutral *oligo-^OX^Ia* conversion to: (**A**) adiabatic radical cation of ^OXO^G_4_:::C_2_ and (**B**) to adiabatic radical anion of ^OX^Ia_2_::C_4_nucleoside pair of *oligo-^OX^Ia*. The depicted distances have been given in [Å], R—2-deoxyribose.

However, the electron affinity at all points was noted to be almost two times higher than that assigned for unmodified double-stranded oligonucleotides (Table 2). The above indicates that oxidized imino-allantoin determines the spot of electron settling, even if the ^OXO^dG is present in close proximity to the system. A systematic *oligo-^OX^Ia* structural analysis revealed that there was a different place/location for the proton transfer (PT) process. A PT was observed between N1(^OXO^G_4_) and N2(C_2_) of^OXO^dG_4_:::C_2_ (a shift of 0.11 [Å])when the ds-oligo lost an electron and formed the cation radical (Figure 3A). The appearance of an excess electron in the system caused radical anion formation and a proton transfer within the oxidized imino-allantoin moiety of ^OX^Ia_2_::C_4_ base pair between N7 and O5 atoms (a shift of 0.14 [Å]), as shown in Figure 3B.

### 2.2. Influence of ^OX^Ia as Part of a CDL on Charge Distribution and Electronic Properties of Basepairs Present in oligo-^OX^Ia

**Figure 4:**
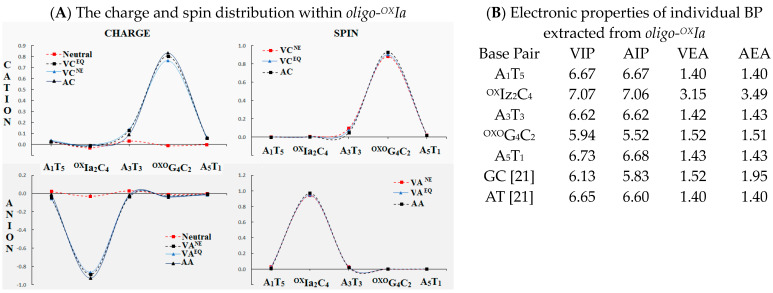
(**A**) A graphical visualization of Hirshfeld charge and spin distribution in [au and %] within neutral, cationic, and anionic forms of *oligo-^OX^Ia* (Table S2 presents the raw data). VC—vertical cation VA—vertical anion, EQ—equilibrated solvent-solute mode, NE—non-equilibrated solvent-solute mode, AC—adiabatic cation, AA—adiabatic anion; (**B**) Electronic properties in [eV] of isolated base pairs from *oligo-^OX^Iz*: vertical (VIP), adiabatic ionization potential (AIP), and vertical (VEA), adiabatic (AEA) electron affinity calculated at the M062x/6-31++G** level of theory in the aqueous phase.

It has been commonly accepted that within a double helix, the positive charge settles on the purine moiety, while the negative one is localized on pyrimidine nucleosides. The results of previous studies have shown that the presence of DNA damage, e.g., ^OXO^dG, strongly influences the pattern of charge distribution. Much less is known, however, about the role of a CDL’s presence. Hence, the ds-pentamer *oligo-^OX^Ia* with a local multi-damage site containing six- and two-electron oxidation dG products, i.e., oxidized imino-allantoin and 7,8-dihydro-8-oxo-guanosine, was taken into theoretical investigation. The non-equilibrated and equilibrated modes of aqueous phase *oligo-^OX^Ia* interaction were used for the charge and spin distribution analysis according to Hirschfeld’s methodology. The formation of a one-electron oxidation spot in the predisposed part of the double helix triggers electron-hole migration through the base pair ladder. This process starts with (A) an interruption of the delicate equilibrium between the solvent and solute, resulting in the ds-DNA adopting a vertical cation non-equilibrated (NE) state (VC^NE^). Subsequently, (B) an equilibrium in the solvent-solute interaction takes place (EQ), and a VC^EQ^ state is formed. This finally converts to (C) ds-oligo geometry rearmament and the appearance of an adiabatic cation (AC). This process was analyzed *step by step* (i.e., VC^NE^→VC^EQ^→AC) in light of the positive charge and spin distribution changes within *oligo-^OX^Ia*. As expected, the presence of an^OXO^G_4_C_2_ base pair within the investigated ds-DNA, irrespective of the presence of ^OX^Ia, was identified as a radical cation sink with the lowest ionization potential (Figure 4). The above is in good agreement with previous results obtained during investigations into isolated DNA damage [37,38].

A different picture emerged when the appearance of an excess electron was investigated. Starting from the initial step, when the vertical anion in a non-equilibrated (VA^NE^) state was generated through solvent-solute equilibration (VA^EQ^) culminating in the formation of an adiabatic anion (AA), the charge and spin were mainly located at the^OX^Ia_2_C_4_ base pairs of *oligo-^OX^Ia*. No additional electron was dispersed over other neighboring BPs, i.e., A_1_T_5_ and A_3_T_3_a, or^OXO^G_4_C_2_ (Figure 4A). This observation correlates well with the results of electron affinity calculations: the highest vertical and adiabatic values were invariably noted for ^OX^Ia_2_C_4_ as follows: 3.15 and 3.49 [eV], respectively (Figure 4B).

### 2.3. The Influence of ^OX^Ia as Part of a CDL on Charge Migration through oligo-^OX^Ia

The results presented above, discussing spatial geometry, charge/spin distribution, and electronic properties of complete *oligo-^OX^Ia* and isolated single base pairs, enable us to predict how the discussed CDL affects excess electron and electron-hole transfer through the double helix. These processes can be discussed in the context of (A) single-step tunneling, (B) random-walk multistep, and (C) polaron-like hopping [39]. According to Marcus’ theory of charge transfer (CT), the process charge moves through the stacked base pair ladder (in an oxidized or reductive state) is characterized by the ***k_CT_*** (rate constant) and the following energetic factors: Δ***G*** (driving force), *λ* reorganization), ***E_a_*** (activation), and ***V_da_*** (electron coupling) [40]. In these studies, the values of all the above parameters were obtained at the M06-2x/6-31++G** level of theory in the condensed phase, as described previously. (For further details, please see reference [41].) Marcus theory was followed in order to investigate the influence of two different lesions derived from the source point of origin (dG), i.e., ^OX^Ia and ^OXO^dG, in the CDL arrangement present in *oligo-^OX^Ia* on the electron-hole/excess migration through the double helix.

As shown in Table 3, the highest *E*_a_ was observed in the case of the electron-hole transfer between ^OX^Ia_2_C_4_→^OXO^G_4_C_2_, i.e., 0.76 [eV], while for the electron transfer between A_1_T_5_←^OX^Ia_2_C_4_, ^OX^Ia_2_C_4_→A_3_T_3_ and ^OX^Ia_2_C_4_←A_5_T_1_, the highest values were, in [eV], as follows: 2.19, 2.05 and 2.04, respectively. Surprisingly, when ^OX^Ia was settled between A_1_T_5_, A_3_T_3_, andA_5_T_1_, the negative values of *E*_a_ were noted, which results in *k*_CT_ being undetectable in the case of radical cation migration. The above indicates that the hole-electron movement between AT pairs through ^OX^Ia pairs should be unaffected and make the transfer towards^OXO^G_4_C_2_ privileged, as shown in Table 3. The migration of additional electrons within the *oligo-^OX^Ia* structure was affected by the presence of ^OX^Ia on account of the electronic properties of ^OX^Ia_2_C_4_ (Figure 4). The *k_CT_* values were found at the level of 10^−21^ s^−1^ for A_1_T_5_→^OX^Ia_2_C_4_, ^OX^Ia_2_C_4_←A_3_T_3_, ^OX^Ia_2_C_4_←^OXO^G_4_C_2_. For the above, a significantly high driving force value was identified (Δ*G* ~ 2 eV), which shifts the charge transfer process to the inverted region. Therefore, CT should be less privileged at these points because of the thermodynamic regime, which is in good agreement with Marcus theory [42].

## 3. Discussion

The ds-DNA structure contained a highly flexible phosphate-sugar skeleton (PS), and an internal rigid BP ladder. The PS can compensate for the fluctuations in geometry forced by the changes in complementary BP geometries. The above stabilized the energy of hydrogen bonds and stacking interactions [43]. It was found that the double helix structure, similar to graphene, is capable of transferring the excess charge over thousands of nucleosides, as shown by Barton et al. [44]. This observation sheds new light on how glycosylasesds-DNA highly effectively scans for lesions, e.g., MutY or Endo III [45]. Both of the above proteins, like other proteins active in the replication and repair of genetic material, contain [4Fe–4S]^2+^ as electron donors [46]. The activity of glycosylases is well known in the context of isolated DNA lesions. The situation becomes more complicated when clustered DNA lesions (CDLs) enter the equation. A CDL is defined as two or more lesions per one or two double-helix turns [47]. This type of damage is especially dangerous and challenging for cell repair systems, which, during lesion removal and repair, try to avoid a potentially lethal double-strand break (DSB) [48].

In the genome, during the oxidizing process, dG can be converted from ^OXO^dG to ^OX^Ia via several “intermediate” products, as shown in Figure 1. These two lesions are formed in the cell in normoxia conditions, while in hypoxia, the ^Fapy^dG formation is preferred [49]. Because of the differences in the structure and electronic properties, ^OXO^dG and ^OX^Ia probably demonstrate a different influence on charge transfer through ds-oligo. In these studies, *oligo-^OX^Ia* (d[A^OX^IaA^OXO^GA]*d[TCTCT]) was investigated at the M06-2x/6-31++G** level of theory in the aqueous phase. Both the non-equilibrated and equilibrated models of solvent-solute interaction and the Hirshfeld model of spin and charge distribution were considered. A comparative spatial geometry analysis revealed that a clustered damage site consisting of ^OX^Ia and ^OXO^G in the *oligo-^OX^Ia* structure had negligible influence according to the standard DNA reference frame parameters (Table 1). Similar to results obtained previously for a ds-DNA fragment containing two ^OXO^G moieties [35], it was noted that the 3D structure was hardly affected at all, contrary to ^OXO^GA mismatch [50].

It should be pointed out that most of the studies on electronic properties have discussed single lesions in the model of double-stranded trimers. Unfortunately, because of the structural regime (terminated base pairs), this approach is inadequate (it fails) in the case of CDL [51,52]. Given this, the role of d[^OX^IaA^OXO^G] in charge transfer through ds-DNA was deemed worth investigating. As commonly accepted, the excess electron or electron hole can migrate through a double helix at a distance greater than 200 [Å] ds-DNA in the form of a radical cation or anion [13].

The loss or adoption of an electron can trigger an electron-hole or electron migration through *oligo-^OX^Ia* through π-stacked base pairs [53]. The results of the theoretical experiments presented in Table 2 show that during vertical cation/anion radical formation, the sugar-phosphate backbone may hardly be involved. This observation tallies with the data obtained for the corresponding native *oligo-N* [50]. Additionally, the recent research of Sevilla et al. shows that for this process, the solute-solvent interaction is crucial at the beginning point (non-equilibrated mode) [54]. The highest difference between the complete *oligo-^OX^Ia* structure and base pair ladder was noted for VEA^NE^, i.e., 2.42 [eV] and 1.82 [eV], respectively.

To shed light on this phenomenon, a Hirshfeld charge and spin calculation was performed. As shown in Figure 4A, the radical cation is mainly located on the^OXO^G_4_C_2_ base pair, regardless of its state—vertical or adiabatic. Moreover, no differences were noted when non-equilibrated and equilibrated solvent-solute interactions were considered. Similar observations were noted for negatively charged *oligo-^OX^Ia*, i.e., the ^OX^Ia_2_C_4_ moiety was noted as the spot of a radical anion in all the discussed cases. Additionally, the above supports well the results of the electronic properties of base pairs isolated from *oligo-^OX^Iz* (Figure 4B). The lowest vertical and adiabatic ionization potential was assigned for ^OXO^G_4_C_2_, i.e., 5.94 and 5.52 [eV], respectively, while the highest absolute value of vertical and adiabatic electron affinity was found for ^OX^Ia_2_C_4_, i.e., 3.15 and 3.49 [eV], respectively. All calculations were performed at the M062x/6-31++G** level of theory in the aqueous phase.

The charge transfer process through ds-DNA, which strictly depends on mutual BP geometry, can be discussed as follows: (A) single-step tunneling, (B) a random-walk multistep, and (C) polaron-like hopping [39,55]. The charge migration over hundreds of angstroms can be discussed in an incoherent manner and depends on the bridge between the donor and acceptor [39]. Conversely, the charge tunneling path can be observed at a distance of only a few base pairs. Even though the above mechanisms are different, the geometry and electronic properties of purine/pyrimidine moieties are crucial. As shown in current and previous results, ^OXO^dG becomes a radical cation sink when present in ds-oligo [56]. Here, it can be assumed that the electron-hole transfer through ^OX^Ia as a bridge should be unaffected (Table 3). Similar results were observed for single-lessoned ds-DNA [57,58]. However, in contrast to the above, it is possible to predict that the ^OX^IaC moiety of the discussed ds-oligo will be the endpoint of the extra electron transfer. As shown in Figure 4, higher values of VEA and AEA as well as spin/charge concentration were obtained for ^OX^IaC. However, the obtained values of driving force (Δ*G*) and activation energy (*E_a_*) of A_1_T_5_→^OX^Ia_2_C_4_, ^OX^Ia_2_C_4_←A_3_T_3_, ^OX^Ia_2_C_4_←^OXO^G_4_C_2_ settled this process in the inverted region (Table 3), which according to Marcus theory, makes the discussed processes unfavorable from a thermodynamic perspective [59]. The following values of *k_CT_* were found: 3.56 × 10^−22^, 6.32 × 10^−20^, and 3.73 × 10^−21^ [s^−1^], respectively. Conversely, a charge transfer parameter analysis revealed that charge migration towards ^OXO^GC within the discussed ds-pentamer should not be affected (Table 3). Therefore, it can be predicted that even if the ^OX^IaC base pair becomes the sink for excess electrons (electronic properties), the charge transfer should be slowed down via a single-step tunneling process. At the same point, the electron transfer towards ^OXO^GC should be privileged, the following *k_CT_* values were noted: 1.05 × 10^14^ and 3.12 × 10^10^ [s^−1^] for A_3_T_3_→^OXO^G_4_C_2_ and ^OXO^G_4_C_2_←A_5_T_1_, respectively. This can manifest as a slowing down of other processes involved in DNA lesion recognition and removal, which can increase the probability of mutagenesis and subsequent carcinogenesis or aging. However, with regard to anticancer therapy (radio/chemo), the presence of ^OX^Ia in the structure of clustered DNA damage can result in improved cancer treatment.

## 4. Materials and Methods

All theoretical experiments were performed as described previously [60]. The structure of the discussed *oligo-^OX^Ia*(d[A_1_^OX^Ia_2_A_3_^OXO^G_4_A_5_]*d[T_5_C_4_T_3_C_2_T_1_]) in neutral, positively (cation), and negatively (anion) charged forms was optimized using DFT (density functional theory) methodology in the condensed phase. The following ds-DNA sequence, containing all four nucleosides, has been previously investigated as a model for this set of studies. The above strategy enabled a comparative analysis of different components contained in the CDL to be completed. Moreover, the distribution of the proposed lesions enabled interference and mutual electronic interaction. The investigated ds-pentamer, which contains 10 bases and 10 deoxyriboses, is a complex system that pushes the limits of the computational resources used. Single geometry optimization to the ground state, at the below-mentioned level, took approximately 40 days (24 CPUs and 48 GB of RAM were dedicated to each process).

### The oligo-^OX^Iageometry Optimization in Condensed Phase

The chosen level of theory, as described below, has previously been carefully investigated and compared with other DFT functionals such as M-11, wB97-XD, and M06-L, for which the following basis set was utilized: 6-31++G*, 6-31++G** and D95** aug-cc-pVDZ Please refer to the supplementary materials for further details [61]. For all the above, Tomasi’s polarized continuum model for solvation was used. The geometry optimization was conducted using ONIOM (our Own N-layered Integrated Molecular Orbital and Molecular Mechanics) methodology [62]. Applying ONION strategy significantly reduces the time and cost of computation, especially when highly flexible units with a high degree of freedom, like deoxyribose, are present. In this study, the flat aromatic nucleobase moieties were described as a high layer and the flexible sugar-phosphate backbone as a low layer, and they were optimized at the M06-2X/D95** and M06-2X/sto-3G levels of theory in the aqueous phase, respectively [63]. It should be mentioned here that the sugar-phosphate backbone does not play a role in charge transfer and can be described as a low-level layer. The negative charges of all the phosphate groups were neutralized by the addition of a proton [64,65,66]. The obtained complete ds-oligo structures in their ground state were converted into a base pair skeleton and were further used for energy calculations. The necessary additional hydrogen atoms were optimized at the M06-2X/D95** level of theory in the aqueous phase.

All energy calculations were performed in the aqueous phase using the M06-2X functional with an augmented polarized valence double-ζ basis set 6-31++G** [63,67], as justified in previous studies [61].The transition dipole moment of excited states and the single point energy calculation were performed using the time-dependent DFT (TD-DFT) method at the M06-2X/6-31++G** level of theory [68,69]. The solvent effect (aqueous phase) was described by applying Tomasi’s polarized continuum model (PCM) [70]. In the PCM model, however, the dielectric constant was used instead of strict H_2_O distribution around ds-DNA. Moreover, the PCM model significantly reduces the required calculation time and accurately describes non-equilibrated solute-solvent interaction. Furthermore, the above enabled the avoidance of random water molecule post positioning, which might not match the native system. Due to the nature of charge transfer, two modes of solvent effect were investigated, i.e., non-equilibrium (NE) and equilibrated (EQ), as described previously [54]. For all optimized geometries, a charge and spin distribution analysis was achieved using the Hirshfeld theory [71]. Generalized Mulliken–Hush theory was used for the electron coupling calculation [72]. The electronic properties, i.e., adiabatic ionization potential (AIP), adiabatic electron affinity (AEA), vertical ionization potential (VIP), and vertical electron affinity (VEA), were calculated as described previously [73].

All calculations were performed on the Gaussian G16 (version C.01) suit [74]. The RMSD values were calculated using BioVia Discovery Studio v20.1.0.19295 software [75].The three-dimensional structural analyses of the investigated ds-DNAs, based on a standard reference frame, were obtained by a 3DNA software package using the web-based interface w3DNA (web 3DNA) [76].

## 5. Conclusions and Further Perspectives

The genome is continuously exposed to harmful factors such as ionization radiation and reactive oxygen/nitrogen species, among others. In the genome, guanine has the lowest ionization potential, which can be oxidized to 7,8-dihydro-8-oxo-2′-deoxyguanosine and subsequently to secondary lesions like ^OX^Ia. ^OXO^dG was intensively studied, unlike Gh, Sp, and ^OX^Ia, on account of its stability and ease of determination using common HPLC methods. The mutagenic potential of ^OXO^G was established at a level of 3% transversion. Under favorable conditions, with a pH above 8.2, 7,8-dihydro-8-oxo-2′-deoxyguanosine can be oxidized to Gh and subsequently converted to ^OX^Ia. This conversion increases the mutagenic potential up to 100%, with a G→C transversion observed.

In this study, the electronic properties and the influence on charge transfer through ds-DNA of the six-electron oxidation product ^OX^Ia were taken into consideration. Unlike its precursor, Gh, which exists in two diastereomers, ^OX^Ia is a non-chiral compound (Figure 1). With this in mind, the d[A_1_^OX^Ia_2_A_3_^OXO^G_4_A_5_]*d[T_5_C_4_T_3_C_2_T_1_] ds-pentamer containing CDLs was investigated and compared with an unmodified analogue (*oligo-N*).

- A structural analysis revealed that a clustered damage site consisting of ^OX^Ia and ^OXO^G in the *oligo-^OX^Ia* structure had negligible influence according to the standard DNA reference frame parameters.
- Theoretical studies revealed that the radical cation prefers to settle on the ^OXO^G moiety, irrespective of the presence of ^OX^Ia in a *ds-oligo*. The lowest vertical (5.94 [eV]) and adiabatic (5.52 [eV]) ionization potential values were found for the ^OXO^G:::C base pair. Conversely, the highest vertical and adiabatic electron affinity was assigned for ^OX^IaC as follows: 3.15 and 3.49 [eV], respectively.
- The Hirshfeld charge and spin analysis indicated that the radical cation is mainly located on the ^OXO^G_4_C_2_ base pair, regardless of its state—vertical or adiabatic. Moreover, no differences were noted when non-equilibrated and equilibrated solvent-solute interactions were considered. Similar observations were noted for negatively charged *oligo-^OX^Ia*, i.e., the ^OX^Ia_2_C_4_ moiety was noted as the spot of a radical anion in all the discussed cases.
- The calculated values of driving force (Δ*G*) and activation energy (*E_a_*) of A_1_T_5_→^OX^Ia_2_C_4_, ^OX^Ia_2_C_4_←A_3_T_3_, and ^OX^Ia_2_C_4_←^OXO^G_4_C_2_ settled this process in the inverted region, which is unfavorable from a thermodynamic perspective. The following values of *k_CT_* were found: 3.56 × 10^−22^, 6.32 × 10^−20^, and 3.73 × 10^−21^ [s^−1^], respectively. At the same point, the electron transfer towards ^OXO^GC should be privileged, and the following *k_CT_* values were noted: 1.05 × 10^14^ and 3.12 × 10^10^ [s^−1^] for A_3_T_3_→^OXO^G_4_C_2_ and ^OXO^G_4_C_2_←A_5_T_1_, respectively.

The above can lead to a slowing down of other processes involved in DNA lesion recognition and removal or genetic material replication, which can increase the probability of mutagenesis and subsequent carcinogenesis or aging. However, with regard to anticancer therapy (radio/chemo), the presence of ^OX^Ia in the structure of clustered DNA damage can result in improved cancer treatment.

### Further Perspectives

DNA damage such as Gh, Sp, ^OXO^G, and ^OX^Ia, when becoming part of clustered DNA damage, can affect the efficiency of the lesion recognition and repair process because it exerts a strong influence on the electron transfer process. Most current studies discuss the CDL in the context of native-type hydrogen bonds (Watson-Crick), while other types like Hoogsteen, reverse Hoogsteen, and Wobble have been investigated to a far lesser degree. Additionally, no studies have been performed on their influence on charge transfer, especially in terms of electron transfer. The same should be pointed out in the case of different ds-DNA forms A, B, and Z. In the context of Barton’s recent studies and the increase in the number of identified proteins involved in DNA repair and replication, an understanding of the influence of CDLs on protein-protein communication is of high scientific value. Investigating this communication process can lead to a greater understanding of the acceleration of harmful/undesirable processes like carcinogenesis or aging. Additionally, it may result in an increase in the efficiency of anticancer radiotherapy/chemotherapy or combined therapies. Therefore, the influence of clustered damage on charge transfer and its subsequent effect on single damage recognition by glycosylases warrants future investigation.

## Figures and Tables

**Table 2:** Electronic properties, in [eV], of *oligo-^OX^Ia*: Vertical (VIP), Adiabatic Ionization Potential (AIP) and Vertical (VEA), Adiabatic (AEA) Electron Affinity calculated at the M062x/6-31++G** level of theory in the aqueous phase. ^(a)^ complete double helix and ^(b)^ base pair skeletons, NE—non-equilibrated solvent-solute interaction, EQ—equilibrated solvent-solute interaction. Root-Mean-Square Deviation (RMSD) of atomic positions in [Å^2^], for Neutral, Anionic and Cationic forms of *oligo-^OX^Ia*. Base pairs (BP), phospho-sugar (PS) frame. The row data have been given in Table S1.

	VIP^NE^	VIP^EQ^	AIP	VEA^NE^	VEA^EQ^	AEA
*oligo-^OX^Ia*	^(a)^ 6.50	5.88	5.49	2.42	3.08	3.59
	^(b)^ 6.30	5.80	5.39	1.86	2.87	3.24
oligo-N*	^(a)^ 6.72	6.08	5.65	0.84	1.58	2.09
	^(b)^ 6.48	5.98	5.58	0.60	1.34	1.90
	RMSD: Anion versus Neutral			RMSD: Cation versus Neutral		
	ds-DNA	BP	PS-Frame	ds-DNA	BP	PS-Frame
*oligo-^OX^Ia*	0.49	0.36	0.59	0.18	0.13	0.22
*oligo-N* [35]	0.17	0.16	0.17	0.36	0.29	0.42

**Table 3:** Charge transfer parameters. The ΔG-driving force [eV], λ-reorganization energy [kcal], *E*_a_-activation energy [eV], *V*_12_-electron coupling, and *k*_CT_-charge rate [s^−1^] constant of permissible transfer between base pairs of *oligo-^OX^Ia*, calculated at the m062x/6-31++G** level of theory in the condensed phase. Arrows indicate the direction of charge migration. The raw data have been given in Tables S3a,b and S4.

Electron-Hole Transfer						Excess-Electron Transfer					
System	*λ*	Δ*G*	*E_a_*	*V*_12_	*K_CT_*	System	*λ*	Δ*G*	*E_a_*	*V*_12_	*k_CT_*
	A_1_^OX^Ia_2_A_3_^OXO^G_4_A_5_					A_1_^OX^Ia_2_A_3_^OXO^G_4_A_5_					
**A_1_T_5_←^OX^Ia_2_C_4_**	−0.01	−0.39	−5.51	0.38	0.00	**A_1_T_5_→^OX^Ia_2_C_4_**	0.35	−2.10	2.19	0.38	3.56 × 10^−22^
**^OX^Ia_2_C_4_→A_3_T_3_**	−0.01	−0.44	−6.27	0.09	0.00	**^OX^Ia_2_C_4_←A_3_T_3_**	0.36	−2.07	2.05	0.32	6.32 × 10^−20^
**A_3_T_3_→^OXO^G_4_C_2_**	0.42	−1.10	0.27	0.43	1.13 × 10^11^	**A_3_T_3_→^OXO^G_4_C_2_**	0.003	−0.08	0.58	0.02	1.05 × 10^14^
**^OXO^G_4_C_2_←A_5_T_1_**	0.35	−1.16	0.46	0.36	5.59 × 10^7^	**^OXO^G_4_C_2_←A_5_T_1_**	0.008	−0.08	0.16	0.01	3.12 × 10^10^
**A_1_T_5_→A_3_T_3_**	0.00	−0.05	−0.16	0.03	0.00	**A_1_T_5_→A_3_T_3_**	0.01	−0.03	0.01	0.07	5.61 × 10^14^
**^OX^Ia_2_C_4_→^OXO^G_4_C_2_**	0.41	−1.54	0.76	0.05	7.04	**^OX^Ia_2_C_4_←^OXO^G_4_C_2_**	0.33	−1.98	2.04	0.06	3.73 × 10^−21^
**A_3_T_3_←A_5_T_1_**	0.00	−0.06	−0.24	0.04	0.00	**A_3_T_3_→A_5_T_1_**	0.00	−0.003	0.01	0.05	5.16 × 10^15^

## Data Availability

Data is contained within the article and Supplementary Materials.

## Supplementary Materials

The following supporting information can be downloaded at: https://www.mdpi.com/article/10.3390/ijms25115962/s1.
