# Supplementary material for: The Influence of Oxidized Imino-Allantoin in the Presence of OXOG on Double Helix Charge Transfer: A Theoretical Approach
Source: Int J Mol Sci. 2024 May 29;25(11):5962. doi: 10.3390/ijms25115962 (PMC11172559; doi:10.3390/ijms25115962)
Supplement: Supplementary file 1 [file ijms-25-05962-s001.zip › Supplementary Materials.pdf]

Supplementary Materials

The influence of oxidized imino-allantoin, in the presence of ^{OXO}G, on double helix charge transfer: a theoretical approach

Bolesław T. Karwowski

Table S1. The energies (in Hartree) of Neural, Vertical Cation (VC^{NC}) (NE-non-equilibrated), Vertical Cation (VC^{EQ}) (EQ-equilibrated), Vertical Anion (VA^{NE}), Vertical Anion (VA^{EQ}), Adiabatic Cation (AC), Adiabatic Anion (AA) of complete DNA double helix and base pairs skeleton extracted from *ds*-oligonucleotides calculated at the M06-2X/6-31++G** level of theory in the aqueous phase, respectively

	**Neutral**	**VC^{NE}**	**VC^{EQ}**	**VA^{NE}**	**VA^{EQ}**	**AC**	**AA**
	**Complete DNA double helix**						
oligo- ^{OX}***Ia***	-12888.6634818	-12888.4245330	-12888.4475184	-12888.7524280	-12888.7766032	-12888.4616365	-12888.7955198
**Base Pairs skeleton extracted from *ds*-oligonucleotides**							
oligo- ^{OX}***Ia***	-4750.4975610	-4750.2661200	-4750.2844040	-4750.5659310	-4750.6031320	-4750.2995830	-4750.6167010

Table S2. Hirshfeld charge and spin distribution in the shape of *oligo-^{OX}Ia* d[$A_1{}^{OX}Ia_2A_3{}^{OXO}G_4A_5$]*d[$T_5C_4T_3C_2T_1$]only nucleosides bases were taken into consideration. calculated at the M06-2x/6-31++G** level of theory in the aqueous phase. Vertical Cation (VC^{NC}) (NE-non-equilibrated). Vertical Cation (VC^{EQ}) (EQ-equilibrated). Vertical Anion (VA^{NE}). Vertical Anion (VA^{EQ}). Adiabatic Cation (AC). Adiabatic Anion (AA)

oligo-^{OX}Ia							
	**Neutral**	**VC^{NE}**		**VC^{EQ}**		**AC**	
	**Charge**	**Charge**	**Spin**	**Charge**	**Spin**	**Charge**	**Spin**
A_1T_5	0.02	0.04	0.00	0.02	0.00	0.03	0.00
**$^{OX}Ia_2C_4$**	-0.03	-0.01	0.00	-0.01	0.00	-0.01	0.00
A_3T_3	0.03	0.14	0.10	0.13	0.07	0.09	0.05
**$^{OXO}G_2C_4$**	-0.01	0.76	0.88	0.81	0.90	0.83	0.93
A_5T_1	0.00	0.07	0.02	0.06	0.02	0.06	0.02
		**VA^{NE}**		**VA^{EQ}**		**AA**	
		Charge	Spin	Charge	Spin	Charge	Spin
A_1T_5		-0.06	0.03	-0.05	0.03	-0.01	0.01
**$^{OX}Ia_2C_4$**		-0.86	0.94	-0.89	0.95	-0.93	0.97
A_3T_3		-0.03	0.03	-0.03	0.03	-0.02	0.02
**$^{OXO}G_2C_4$**		-0.03	0.00	-0.03	0.00	-0.04	0.00
A_5T_1		-0.02	0.00	-0.01	0.00	-0.01	0.00

Table S3a. The Energies: Ground (E^{GR}) and Excitation (E^{EX}) state energies and Excitation and HOMO Energies as well as corresponding Dipole Moments Ground. Excitation. and Transition (DM^{G}. DM^{EX}. D_{12}) in Debays of neighbor base pair extracted from selected dimmers of *oligo-^{OX}Ia*. calculated at the M06-2x/6-31++G** level of theory in the aqueous phase using the DFT or TD-DFT methodology.

SYSTEM	B.P. Dimer	E^{GR}	DM^{GR}	E^{EX}	DM^{EX}	D_{12}	E^{HOMO}	E^{HOMO-1}	E^{LUMO}	E^{LUMO+1}
oligo-^{OX}Ia	**A_1ll $^{OX}Ia_2$**	-1895.555617	13.84	-1895.442378	15.37	0.37	-0.2961	**0.011933**	-0.0735	-0.0175
	**$^{OX}Ia_2$llA_3**	-1895.557571	14.43	-1895.444388	16.73	1.23	-0.2814	-0.2971	-0.0728	-0.0147
	**A_3ll$^{OXO}G_4$**	-1933.696352	16.90	-1933.571578	15.24	3.67	-0.2512	-0.2833	-0.0163	-0.0151
	**$^{OXO}G_4$llA_5**	-1933.693705	16.39	-1933.566309	14.79	1.74	-0.2545	-0.2838	-0.0178	-0.0139

Table S3b. The Energies: Ground (E^{GR}) and Excitation (E^{EX}) state energies and Excitation and HOMO Energies as well as corresponding Dipole Moments Ground. Excitation. and Transition (DM^{G}. DM^{EX}. D_{12}) in Debays of distal base pair extracted from selected trimmers of *ds*-oligonucleotides. calculated at the M06-2x/6-31++G** level of theory in the aqueous phase using the DFT or TD-DFT methodology

SYSTEM	Base Pair Dimer	E^{GR}	DM^{GR}	E^{EX}	DM^{EX}	D_{12}	E^{HOMO}	E^{HOMO-1}	E^{LUMO}	E^{LUMO+1}
oligo-^{OX}Ia	**A_1llA_3**	-1895.555617	13.83502	-1895.442378	15.36593	0.43	-0.284195	-0.296128	-0.073512	-0.017476
	**$^{OX}Ia_2$ll$^{OXO}G_4$**	-1895.557571	14.42834	-1895.444388	16.73471	1.23	-0.281432	-0.29705	-0.072813	-0.014694
	**A_3llA_5**	-1933.696352	16.90233	-1933.571578	15.2382	3.67	-0.251187	-0.283261	-0.016303	-0.015124

Table S4. The energies (in Hartree) of Neural. Vertical Cation. Adiabatic Cation forms of base pairs extracted from *oligo-^{OX}Ia* calculated at the M06-2x/6-31++G** level of theory in the aqueous phase.

oligo-Iz	**Neutral**	**Vertical Cation**	**Adiabatic Cation**
**A_1T_5**	-921.192272	-920.947272	-920.947128
**$^{OX}Ia_2C_4$**	-974.339582	-974.079773	-974.080108
**A_3T_3**	-921.192413	-920.94922	-920.949047
**$^{OXO}G_4C_2$**	-1012.477062	-1012.258818	-1012.274182
**A_5T_1**	-921.192199	-920.944997	-920.946701
		**Vertical Anion**	**Adiabatic Anion**
**A_1T_5**		-921.24366	-921.243537
**$^{OX}Ia_2C_4$**		-974.455328	-974.467955
**A_3T_3**		-921.244712	-921.244874
**$^{OXO}G_4C_2$**		-1012.532978	-1012.532614
**A_5T_1**		-921.244864	-921.244788
